# Fit accuracy in the rest region of RPDs fabricated by digital technologies and conventional lost-wax casting: a systematic review and meta-analysis

**DOI:** 10.1186/s12903-023-03348-6

**Published:** 2023-09-15

**Authors:** Jiachao Qiu, Wei Liu, Danfeng Wu, Feng Qiao, Lei Sui

**Affiliations:** 1 Department of Prosthodontics, Tianjin Medical University School and Hospital of Stomatology, Qixiangtai Road No.12, Tianjin, 300070 P.R. China; 2 Department of Stomatology, North Sichuan Medical College, Affiliated Hospital of North Sichuan Medical College, Nanchong, 637000 People’s Republic of China; 3 Department of Oral and Maxillofacial Surgery, Tianjin Medical University School and Hospital of Stomatology, Qixiangtai Road No.12, Tianjin, 300070 P.R. China

**Keywords:** Removable partial denture, Rest, Digital technology, Conventional lost-wax casting, Fit accuracy

## Abstract

**Background:**

Digital technologies have recently been introduced into the fabrication of removable partial dentures (RPDs). However, it is still unclear whether the digitally fabricated RPDs fit better than conventionally cast ones in the rest region. The aim of this study was to evaluate the fit accuracy in the rest region of RPDs fabricated by digital technologies and compare it to those made by conventional lost-wax (CLW) technique.

**Methods:**

A comprehensive search was conducted in Cochrane Library, PubMed, EMbase, Web of Science and SpringerLink. Studies published up to August 2022 were collected. Two authors analyzed the studies independently and assessed the risk of bias on the modified methodological index for non-randomized studies (MINORS) scale. The mean values of gap distance between rests and corresponding rest seats of each study were extracted as outcome. A random-effects model at a significance level of *P* < 0.05 was used in the global comparison and subgroup analysis was carried out.

**Results:**

Overall, 11 articles out of 1214 complied with the inclusion criteria and were selected, including 2 randomized controlled trials (RCTs), 1 non-randomized clinical trial and 8 in vitro studies. Quantitative data from Meta-analysis revealed that fit accuracy in the rest region of RPDs fabricated with CLW showed no statistically significant difference with digital techniques (SMD = 0.33, 95%CI (-0.18, 0.83), *P* = 0.21). Subgroup analysis revealed a significantly better fit accuracy of CLW-fabricated RPDs in the rest region than either additive manufacturing (AM) groups or indirect groups (*P* = 0.03, *P* = 0.00), in which wax or resin patterns are milled or printed before conventional casting. While milled RPDs fit significantly better than cast ones in the rest region (*P* = 0.00). With digital relief and heat treatment, hybrid manufactured (HM) clasps obtained better fit accuracy in the rest region (*P* < 0.05). In addition, finishing and polishing procedure had no significant influence in the fit accuracy in all groups (*P* = 0.83).

**Conclusions:**

RPDs fabricated by digital technologies exhibit comparable fit accuracy in rest region with those made by CLW. Digital technologies may be a promising alternative to CLW for the fabrication of RPDs and additional studies are recommended to provide stronger evidence.

**Trial registration:**

CRD42020201313.

**Supplementary Information:**

The online version contains supplementary material available at 10.1186/s12903-023-03348-6.

## Introduction

In traditional dental practice, removable partial dentures (RPDs) are fabricated by lost-wax casting [1]. By this collaborative process between dentists and dental technicians, high-quality RPD frameworks can be produced. Nevertheless, it requires a great deal of experience and remains to be labor-intensive [2]. In 1970s, computer-aided design/computer-aided manufacturing (CAD/CAM) was applied into dentistry by Duret and Preston [3]. Since then, digital technology began its dental life.

A CAM system can be categorised into two types: subtractive manufacturing (SM) and additive manufacturing (AM). The most common SM technology used in dentistry is computer numerical controlled (CNC) milling. This method uses a milling machine to produce the object by removing bulk material from solid blocks with all the steps controlled by a computer program [4, 5]. While AM, also known as three-dimensional printing or rapid prototyping (RP), includes a range of different technologies such as stereolithography (SLA), selective laser melting (SLM), selective laser sintering (SLS), direct metal laser-sintering (DMLS), fused deposition modeling (FDM), selective electron beam melting (SEBM) and inkjet printing [4, 5]. SLA is basically used in the manufacture of resin-based structures, for instance temporary crowns, acrylic teeth, dentures, mouth guard and bite plane appliances through deposition of consecutive layers of photosensitive material that is readily polymerized [6, 7]. SLM, SLS and DMLS are laser powder forming techniques that use a high-energy laser beam to fuse material in its powder form and construct 3D objects layer by layer [6]. When processing polymers and ceramic the industry generally refers to this as SLS whereas for metals the terms used are SLM or DMLS [5]. FDM is a filament extrusion-based process that a plastic filament is heated to a semiliquid state and then extruded through a nozzle to deposit on to a platform to create 3D parts directly from a CAD model [5, 8]. SEBM is generally used for forming near-net shaped components of metals by melting metal powder layer per layer with an electron beam in a high vacuum [5, 9]. Inkjet printing works by propelling individual small ink drops toward a substrate and is capable of printing objects using two materials with quite distinctively different properties [5].

Milling(MI) manufacturing is superior in creating a smooth surface, while AM technologies overcome the limitations of subtractive methods, producing complex small shapes layer by layer directly from a computer model without limitations of the size of the smallest cutting tool [5, 10]. And thus hybrid manufacturing(HM) emerged, combining the advantages of the two techniques. Nakata et al. developed a one-process molding machine which integrated repeated laser sintering and MI into a single platform [11, 12]. Due to economic considerations, the indirect digital method consists of milling or printing wax/resin patterns that are then converted into cast-metal frameworks through conventional lost-wax technique (CLW) [13]. All these technologies mentioned above are collectively referred to as digital technologies in this review.

It should be a primary quality of CAD/CAM systems that they can produce accurate fitting prosthetic components [14]. The Aker’s clasp commonly used in RPDs is composed of three parts: clasp arm, counter arm and the rest. Rests affords efficient resistance to functional chewing forces, which are transmitted vertically to the abutment teeth and conducted along the long axes of the teeth. To avoid independent movement or slippage of RPDs under occlusal loading, the rests and the teeth must remain in stable contact. Considering this, it is important for the rests to not only be rigid but also fit accurately to the rest seats [15]. Stern et al. evaluated the adaptation between the occlusal rests and their corresponding rest seats in order to investigate the clinically acceptable in the fit accuracy of RPDs [16]. Fit accuracy of digitally fabricated RPD rests have been evaluated and described in several studies [11–13, 17–24], with inconsistent conclusions. In a study by Pelletier et al., frameworks made with SLS were less accurate at rest region than those produced with CLW [23], while Soltanzadeh et al. found that compared to 3D-printed groups, the cast RPD group showed better overall fit and accuracy [24]. Therefore, it is still unknown whether the digital technologies could provide acceptable fit accuracy for the rests in RPDs.

The purpose of this study is to systematically review in vitro and clinical studies comparing the fit accuracy in the rest region of RPDs fabricated by digital technologies and conventional lost-wax technique. The null hypothesis was that no differences would be found between CLW and digital technologies.

## Methods

### Search methods

This systematic review was registered in the International Prospective Register of Systematic Reviews (PROSPERO: CRD42020201313). A systematic approach was followed according to the Preferred Reporting Items for Systematic Review and Meta-Analyses (PRISMA) Statement [25] and the Cochrane Handbook [26]. The search strategy was based on the PICOS (Population, Intervention, Comparison, Outcomes, Study) format:

- P (Population): Removable Partial Denture
- I (Intervention): Digital technologies including AM (3D printing etc.), SM (MI etc.), indirect digital technologies (milling or printing of wax/resin patterns followed by CLW) and HM.
- C (Comparator): Conventional lost-wax casting technology
- O (Outcome): Fit accuracy in the rest region, which is represented by the gap distances between the rest seats and the intaglio surfaces of the occlusal rests (μm)
- S (Study): Clinical studies and in vitro studies

An electronic search was performed in Cochrane Library, PubMed, EMbase, Web of Science and SpringerLink on August 2nd, 2022, including articles published from January 1950 until August, 2022. No publication language restrictions were taken into account (Table 1). We used Medical Subject Headings (MeSH) terms and EMTREE, along with free-words to target the PICOS. In addition to the electronic search, relevant reviews and references lists of included full-text articles were manually checked as well.

**Table 1:** Full search strategies for all databases

Databases	Full search strategies
Cochrane library	"removable partial denture" in All Text AND "CAD/CAM" OR "Computer-Aided Design" OR "Computer-Aided Manufacturing"OR" Computer-Assisted Design" OR" Computer-Assisted Manufacturing"OR"3-D Printing"OR"3-Dimensional Printing"OR"3D Printing"OR"Three-Dimensional Printing"OR"milling"OR"milled"OR"additive manufacturing technologies" OR"additive manufacturing"OR"digital workflow"OR "digital technology" OR "computer* dentistry" OR "virtual design" OR "rapid prototyping" OR "rapid manufacturing" OR "RP techniques" OR "manufacturing" in All Text—(Word variations have been searched)
Web of Science	("Removable Partial Denture"OR”clasp”)AND( "CAD/CAM" OR "Computer-Aided Design" OR "Computer-Aided Manufacturing"OR" Computer-Assisted Design" OR" Computer-Assisted Manufacturing"OR"3-D Printing"OR"3-Dimensional Printing"OR"3D Printing"OR"Three-Dimensional Printing"OR"milling"OR"milled"OR"additive manufacturing technologies" OR"additive manufacturing"OR"digital workflow"OR "digital technology" OR "computer* dentistry" OR "virtual design" OR "rapid prototyping" OR "rapid manufacturing" OR "RP techniques" OR "manufacturing")
PubMed	(("Removable Partial Denture"OR”clasp”) AND ("CAD/CAM” OR “Computer-Aided Design” OR “Computer-Aided Manufacturing”OR” Computer-Assisted Design” OR” Computer-Assisted Manufacturing”OR”3-D Printing”OR”3-Dimensional Printing”OR”3D Printing”OR”Three-Dimensional Printing”OR”milling”OR”milled”OR”additive manufacturing technologies” OR “additive manufacturing”OR”digital workflow”OR “digital technology” OR “computer* dentistry” OR “virtual design” OR “rapid prototyping” OR “rapid manufacturing” OR “RP techniques” OR “manufacturing”))
EMbase	('removable partial denture'/exp OR 'rpd (denture)' OR 'swing-lock' OR 'denture, partial, removable' OR 'partial denture, removable' OR 'partial dentures, removable' OR 'removable partial denture' OR 'removable partial dentures' OR clasp) AND ('computer aided design/computer aided manufacturing'/exp OR 'cad/cam software'/exp OR 'three dimensional printing'/exp OR '3 dimensional printing' OR '3-d printing' OR '3d printing' OR '3dp additive manufacturing' OR 'additive layer manufacturing' OR 'printing, three-dimensional' OR 'three dimensional printing' OR 'three-dimensional printing' OR 'milling'/exp OR 'additive manufacturing' OR 'rapid prototyping'/exp OR 'rapid proto-typing' OR 'rapid prototyping' OR 'digital workflow')
SpringerLink	(Removable Partial Denture) AND (CAD/CAM OR 3D printing OR milling* OR computer* dentistry)

### Screening and selection criteria

Studies that reported outcome data for both digitally and conventionally fabricated RPDs or clasp samples were included. All related studies with an English abstract were included in this review. For control group, only RPDs fabricated by CLW were included. RPDs fabricated by digital technologies, including AM, SM, HM and indirect digital technologies were included as intervention group.

The main outcome for this review was fit accuracy in the rest region, which was defined as the gap distance in micrometers between the rests and their corresponding rest seats. Randomized controlled trials (RCT), non-randomized clinical studies and in vitro studies were included. Case reports, case series, expert opinions, commentaries, editorials, reviews, and conference abstracts were excluded. A study that was not accessible to read in full or was not available in the databases was also excluded (Table 2).

**Table 2:** Inclusion and exclusion criteria

Inclusion Criteria
1. Study types: Randomized controlled trials (RCT), non-randomized clinical trials, cohort studies, case control studies and other observational studies as well as in vitro studies
2. Articles published in the period from 1950 until Aug. 2022
3. No publication language limits taken into account
Exclusion Criteria
1. Articles that used only qualitative method to evaluate the fit accuracy, such as clinical check and pressing test without available data
2. Articles that studied the RPDs fabricated by indirect digital technology (printing or milling wax/resin patterns before investment casting) rather than CLW as control groups
3. Reviews and studies with only charts and questionnaires
4. Articles unavailable in the databases or articles that are inaccessible to read in full

A reference manager software program (EndNote v.X9.3.1) was used and the duplicates were discarded electronically. The remaining articles derived from the extensive search were screened through title and abstract by two reviewers (JQ, DW) independently. The full-text was checked if title and abstract provided insufficient information with regards to the inclusion criteria. Finally, articles selected from the inclusion and exclusion criteria were further screened in full-text and double-checked by both reviewers (JQ, DW). Any disagreements at the above stages between reviewers were resolved by consulting a third reviewer (LS) and discussion until consensus was reached.

### Data extraction

Two authors (JQ and DW) conducted the data extraction as well as risk of bias assessments independently, and any disagreements were resolved through consensus. The following information was extracted: 1) Author and year of publication; 2) Study design; 3) Groups; 4) Tooth die or model type; 5) Sample type; 6) Method used for evaluating the fit accuracy of the rest); 7) Sample size; 8) Main outcomes; 9) Scanning information; 10) CAD software; 11) Manufacturing machine; 12) Finishing and polishing. We contacted the corresponding authors of individual studies for missing data or additional study information. And those with no respondence after three contact attempts were excluded from meta-analysis and included in the qualitative aspect of this review. For studies that reported the gap distance values before and after polishing of the samples, the data after polishing was selected for the global meta-analysis. And for studies that evaluated vertical and horizontal distances between the RPD rests and rest seats, only the horizontal data (distances between the bottom of rests and rest seats on the occlusal surface of the tooth) was extracted.

### Critical appraisal

The Cochrane Risk of Bias Assessment Tool for Randomized Controlled Trials [26] were used with the software RevMan version 5.3 (The Cochrane Collaboration, Copenhagen, Denmark) to assess the risk of bias for two included RCTs [17, 23]. And to assess the risk of bias of other included in vitro experiments [11–13, 19–22, 24] and a non-randomized clinical study [18], we developed a modified version of Methodological Index for Non-Randomized Studies (MINORS) scale based on the original one [27]. A total of 13 items were included in the adapted scale, with an additional item proposed for clinical studies (Table 3). The items are scored 0 (not reported), 1 (reported but inadequate) or 2 (reported and adequate) [27]. Discrepancies of opinion during the assessment were resolved through discussion until a consensus was finally reached between the 2 reviewers (JQ and DW). And finally the overall score was calculated. The ideal global score would be 24 for the in vitro studies and 26 for the clinical studies.

**Table 3:** Modified version of MINORS scale

Methodological index for included studies	Scores & Standards
1. A clearly stated aim	0: not reported, 1: reported but inadequate, 2: reported and adequate
2. Impression or scanning method	0: not reported, 1: reported but inadequate, 2: reported and adequate
3. Manufacturing method	0: not reported, 1: reported but inadequate, 2: reported and adequate
4. Abutment	0: not reported, 1: master die/tooth model, 2: natural tooth
5. Prospective collection of data	0: not reported, 1: reported but inadequate, 2: reported and adequate
6. Criteria used to evaluate fit accuracy	0: not reported, 1: reported and using clinical check criteria, 2: reported and fit accuracy is defined as the gap distance between the rest and rest seat area
7. Adequate number of measurement points per specimen	0: not reported, 1: reported but less than 10 points, 2: reported and more than 10 points
8. An adequate control group	0: not reported, 1: reported and adequate compared to other digital methods 2. reported and adequate compared to CLW
9. Contemporary groups	0: not reported, 1: reported but inadequate, 2: reported and adequate
10. Unbiased assessment of the gap distances	0: not reported, 1: reported and measured by a single operator, 2: reported and measured with blinding by a single operator or using surface-matching software program
11. Prospective calculation of the study size	0: not reported, 1: reported but inadequate, 2: reported and adequate
12. Adequate statistical analysis	0: not reported, 1: reported but inadequate, 2: reported and adequate
Additional criteria for included clinical studies	
13. Baseline equivalence of groups	0: not reported, 1: reported but inadequate, 2: reported and adequate

### Statistical analysis

A software program StataMP17.0 was used for data processing and meta-analysis. The number of rests was considered as a statistical unit. Standardized mean difference (SMD) with 95% confidence interval (95% CI) was used to compare digital technologies and CLW fabricated RPDs on fit accuracy in the rest region. The Dersimonian-Laird method was used in the random effects model and the Inverse-variance method was used in the fixed effects model to account for differences between studies. Using Cochrane Q test and I^2^ test (25–50% slight, 50–75% moderate, and > 75% high heterogeneity), heterogeneity among the pooled studies was tested [28, 29]. The *P* < 0.05 was considered statistically significant. According to different digital technologies adopted by each experimental group (AM, MI and indirect digital technologies), the included studies were assigned to three subgroups and subgroup analysis was conducted to investigate possible causes of heterogeneity among the results. The final results were presented by forest maps. And to assess robustness of the synthesised results, sensitivity analyses were conducted by excluding the remaining articles into the literature one by one, conducting meta-analysis again and comparing them with the overall results before exclusion. Potential publication bias among studies included in the meta-analysis were assessed and presented by Funnel plots. In order to reduce the risk of bias in our reference list and avoid any risk of auto-citation read, the fi-index tool was used [30, 31]. For the research results that cannot be integrated, a comprehensive description and separate analysis was carried out.

## Results

### Search and selection

**Fig. 1:**
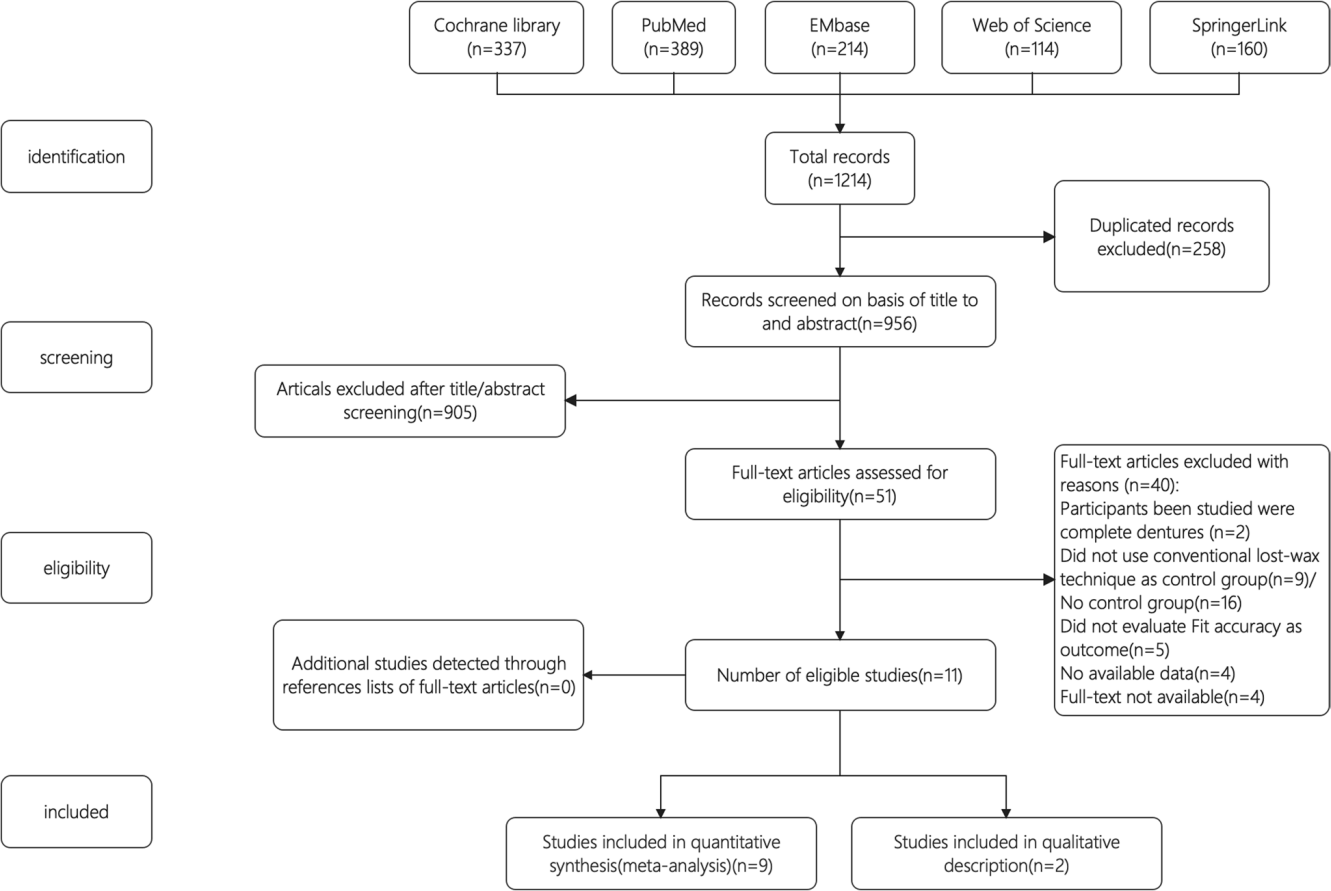
Flow chart of study selection process according to PRISMA diagram

The final electronic search identified 1214 database articles, 337 from Cochrane Library, 389 from PubMed, 214 from EMbase, 114 from Web of Science and 160 from SpringerLink. After removal of duplicates, 956 records remained, from which 905 were excluded through screening on the basis of titles and abstracts. And the remained 51 articles were read in full. Forty publications were further excluded as they did not meet inclusion criteria or lack of available data, leaving 11 as eligible studies for this systematic review. Details of the selection process are presented in Fig. 1.

### Study characteristics

Among the included 11 studies, one was a double-blind, crossover designed RCT [17]. One was a triple-blinded RCT [23], and another a non-randomized clinical study [18]. The remaining 8 were in vitro studies [11–13, 19–22, 24]. All of the studies were published in English. The earliest study was published in 2017 [11] and the most recent was in 2022 [17, 21–23].

Nine studies had RPD frameworks as the unit of analysis, while two studies used Akers clasp assemblies [11, 12]. Regarding the fabrication materials, cobalt-chromium (Co-Cr) was most commonly used, while one study made one-piece RPDs from polyetheretherketone (PEEK) [19] and another one cast clasp samples of CP titanium Grade 3 [11].

The manufacturing techniques included SLM, SLS, DMLS, MI, indirect digital technologies and HM (Repeated laser sintering (RLS) and MI). 7 studies compared the fit accuracy of CLW fabricated RPDs with selective laser melted ones [13, 17, 18, 20–22, 24]. 2 studies evaluated fitness on the rest region of PEEK RPDs fabricated by milling as compared to CLW RPDs [13, 19]. And another two focused on the comparison between hybrid manufactured (RLS and MI) and conventionally cast Akers clasps [11, 12]. As regard to the indirect digital technologies, 3 studies printed resin models for investment and casting [21, 22, 24] while one printed wax patterns for CLW [13]. And in 2 studies wax frameworks were milled and cast [13, 21].

All studies evaluated the horizontal gap between the rests and rest seats, while one addressed the gap distance from both horizontal and vertical dimensions [13]. There’s no standard method for the quantitative measurement of fit accuracy of RPD rests. Among the included studies, 6 used the silicone film method and 2 applied silicone film method combined with digital superimposition to evaluate the fit accuracy [19, 22]. One study scanned the intaglio surface of each RPD framework and superimposed the STL file onto that of its master model [24] and another 1 made the measurement directly under a light microscopy at × 560 magnification [13]. In addition, clinical observation including visual inspection and pressing test were also conducted by three studies [17–19]. Detailed information of individual studies is presented in Tables 4, 5 and 6.

**Table 4:** Summary of included studies (basic information)

Author Year	Study Design	Control group	Study Group	Sample Type
Chia et al., 2022 [17]	RCT (double-blind, crossover)	CLW	SLM	RPD framework (Co-Cr)
Pelletier et al., 2022 [23]	RCT (triple-blinded)	CLW	SLS	RPD framework metal
Ye et al., 2017 [18]	non-randomized clinical study	CLW	CAD/RP(SLM)	RPD framework (Co-Cr)
Ye et al., 2018 [19]	in vitro	CLW	MI(PEEK)	RPD framework (PEEK)
Soltanzadeh et al., 2019 [24]	in vitro	CLW	CAD/RP: SLM CAD/RPS: SLM from stone model LWTR: CLW from printed resin model	RPD framework (Co-Cr)
Bajunaid et al., 2019 [20]	in vitro	CLW	SLM	RPD framework (Co-Cr)
Arnold et al., 2018 [13]	in vitro	CLW	(Rpi) 3D-printing (wax) + CLW (RPd) SLM (MIi) MI (wax) + CLW (MId) MI (PEEK)	RPD framework (Co-Cr)
Oh et al., 2022 [22]	in vitro	CLW	(MEP group) 3D-printing (RPC group) 3D-printing (resin) + CLW	RPD framework (Co-Cr)
Muehlemann et al., 2022 [21]	in vitro	CLW	(C-M) MI + CLW (C-P) 3D-printing (resin) + CLW (SLM) SLM (DMLS) DMLS	RPD framework (Co-Cr)
Torii et al., 2018 [12]	in vitro	CLW	HM (RLS + MI) RLS	Akers clasp (Co-Cr)
Nakata et al., 2017 [11]	in vitro	CLW	HM (RLS + MI)	Akers clasp (cast Co-Cr and CP Ti clasp /CAM Co-Cr clasp)

*RCT* Randomized controlled trials, *CLW* Conventional lost-wax technique, *SLM* Selective laser melting, *RPD* Removable partial denture, *SLS* Selective laser sintering, *CAD/RP* Computer-aided design/ Rapid prototyping, *MI* Milling, *PEEK* Poly ether ether ketone, *CAD/RPS* Selective laser melting from stone model, *LWTR* Lost-wax technique from resin model, *Rpi* Indirect rapid prototyping, *Rpd* Direct rapid prototyping, *MIi* Indirect milling, *MId* Direct milling, *MEP* Metal 3D printing, *RPC* Resin printing and subsequent casting, *C-M* Conventional casting of milled sacrificial patterns, *C-P* Conventional casting of printed sacrificial patterns, *DMLS* Direct metal laser-sintering, *HM* Hybrid manufacturing, *RLS* Repeated laser sintering

**Table 5:** Summary of included studies (measurement information)

Author Year	Model/die	Measurements of fit accuracy	Sample size	Main outcomes
Chia et al., 2022 [17]	29 participants 11 of Kennedy class I or II 18 of Kennedy class III or IV	1.visual gap inspection 2.Silicone film method	*n* = 29	SLM:273.7 ± 44.5 μm Traditional: 242.2 ± 44.5 μm linear mixed-effect model (*P* = .250)
Pelletier et al., 2022 [23]	18 participants	Silicone film method	*n* = 28 (SLS) *n* = 31 (CLW)	SLS: 398 ± 45 µm CLW: 176 ± 41 µm
Ye et al., 2017 [18]	15 patients with dentition defects	1.Visual inspection + Pressing test 2.Silicone film method	*n* = 40	CAD/RP:174 ± 117 µm CLW:108 ± 84 µm Paired t test (*P* = .003)
Ye et al., 2018 [19]	A standard stone cast of a partially edentulous mandible	1.Visual inspection + Pressing test 2.Silicone film method + 3D digital analyses	*n* = 45	PEEK:86.2 ± 22.6 µm Traditional:133.9 ± 49.7 µm Independent samples *t* test (*P* = .003)
Soltanzadeh et al., 2019 [24]	Maxillary Kennedy class III modification I	Surface-matching	*n* = 40	Group I(LWT): -0.02 ± 0.02mm Group II(CAD/RP): 0.03 ± 0.03mm Group III(CAD/RPS): 0.003 ± 0.02mm Group IV(LWTR): -0.032 ± 0.01mm
Bajunaid et al., 2019 [20]	Mandibular Kennedy class III modification I	Silicone film method	*n* = 60	CLW:279.61 ± 175.21 μm SLM:272.16 ± 173.55 μm independent t-test (*P* > 0.05)
Arnold et al., 2018 [13]	Maxilla kennedy I modification III	Observed directly using light microscopy at × 560 magnifification	*n* = 12	LWT:133 ± 59μm Rpi:323 ± 188 μm RPd:365 ± 205 μm ΜIi:117 ± 34 μm ΜId:43 ± 23 μm
Oh et al., 2022 [22]	Maxillary Kennedy Class II, modification 1	Silicone film method + digital superimposition	*n* = 30	CON group: 240.12 ± 64.99 µm MEP group: 211.91 ± 16.84 µm RPC group: 259.26 ± 45.41 µm One-way repeated-measures analysis of variance
Muehlemann et al., 2022 [21]	Mandibular Kennedy Class II, modification 2	Silicone film method	*n* = 3	CLW: 425.59 ± 147.59 µm SLM: 482.93 ± 239.24 µm DMLS: 410.26 ± 79.94 µm C-M: 398 ± 36.35 µm C-P: 600.89 ± 193.03 µm
Torii et al., 2018 [12]	A tooth die simulating the first molar	Silicone film method	*n* = 20	HM: 73.9 ± 1.6 μm RLS: NR CAST: 123.8 ± 2.93 μm
Nakata et al., 2017 [11]	A tooth die simulating the first molar	Silicone film method	*n* = 15	Gap distances(rest): CAST Co-Cr:123.8 ± 2.93 μm CAST CP Ti:130.5 ± 1.80 μm CAM Co-Cr:167.4 ± 9.47 μm

*SLM* Selective laser melting, *SLS* Selective laser sintering, *CLW* Conventional lost-wax technique, *CAD/RP* Computer-aided design/ Rapid prototyping, *LWT* Lost-wax technique, *CAD/RPS* Selective laser melting from stone model, *LWTR* Lost-wax technique from resin model, *Rpi* Indirect rapid prototyping, *RPd* Direct rapid prototyping, *ΜIi* Indirect milling, *ΜId* Direct milling, *CON* Conventional lost-wax technique, *MEP* Metal 3D printing, *RPC* Resin printing and subsequent casting, *DMLS* Direct metal laser-sintering, *C-M* Conventional casting of milled sacrificial patterns, *C-P* Conventional casting of printed sacrificial patterns, *HM* Hybrid manufacturing, *RLS* Repeated laser sintering, *NR* Not reported, *Co-Cr* Cobalt-chromium

**Table 6:** Summary of included studies (manufacturing information)

Author Year	Scanning information	CAD software	Manufacturing machine	Finished and polished
Chia et al., 2022 [17]	lab scanner (D800, 3Shape A/S)	Dental system 2018; 3Shape A/S	SLM RP system (M270; EOS)	YES
Pelletier et al., 2022 [23]	NR	NR	NR	YES
Ye et al., 2017 [18]	lab scanner (D800, 3Shape)	Dental System, 3Shape	SLM RP system (M270, EOS)	YES
Ye et al., 2018 [19]	lab scanner (D800, 3Shape)	Dental System 2015, 3Shape (framework and artificial teeth design) Geomagic Studio 2012, Geomagic (denture bases design and 3D digital analyses)	five-axis milling machine (Organical Multi, R + K)	NR
Soltanzadeh et al., 2019 [24]	TRIOS 3 intraoral scanner (3Shape North America)	RPD designing software (3Shape Removable Partial Design; 3Shape North America)	NR	NO
Bajunaid et al., 2019 [20]	Optical structured-light Scanner S600 ARTI (Zirkonzhan, South Tyrol, Italy)	3 Shape dental software systems, Copenhagen, Denmark	rapid prototyping machine (Mlap Cusing Machine fiber laser100 W(cw), Concept Laser, Germany	YES
Arnold et al., 2018 [13]	D900 scanner; 3Shape A/S)	CAD-CAM software (3Shape-Dental Designer 2013 v2.8.8; 3Shape A/S)	RPd: CNC Construction mlab: M1 cusing (Concept Laser GmbH) MId: 5-axis milling-machine: Organical D7C (R + K CAD-CAM Technologie GmbH & Co. KG)	YES
Oh et al., 2022 [22]	tabletop scanner T500, Medit Co., Seoul, Korea	CAD software (Dental System version 19.1.0, 3Shape A/S, Copenhagen, Denmark)	Mlab cusing 200R GE Additive (Concept Laser)	YES
Muehlemann et al., 2022 [21]	NR	RPD designing software (SilaPart CAD; Siladent)	C-M: CNC Milling machine (In Lab ML X5; Sirona) C-P: 3D printer (Eden 260V; Stratasys 3D-Printer) SLM: direct metal laser melting machine (Mlab cusing; Concept Laser) DMLS: direct metal laser melting machine (ProX DMP 100 Machine; 3D Systems)	YES
Torii et al., 2018 [12]	lab scanner (7 Series, Dental Wings, Montreal, Canada)	CAD system (DWOS Partial Frameworks, Dental Wings)	One-process molding (LUMEX Advance-25, Matsuura Machinery Corp., Fukui, Japan)	NO
Nakata et al., 2017 [11]	lab scanner (7 Series, Dental Wings, Montreal, Canada)	CAD system (DWOS Partial Frameworks, Dental Wings)	One-process molding (LUMEX Advance-25, Matsuura Machinery Corp., Fukui, Japan)	NO

*CAD* Computer-aided design, *SLM* Selective laser melting, *RP* Rapid prototyping, *NR* Not reported, *CAD-CAM* Computer-aided design—Computer-aided manufacturing, *RPd* Direct rapid prototyping, *MId* Direct milling, *RPD* Removable partial denture, *C-M* Conventional casting of milled sacrificial patterns, *C-P* Conventional casting of printed sacrificial patterns, *DMLS* Direct metal laser-sintering

### Results of individual studies

#### Risk of bias in studies

**Fig. 2:**
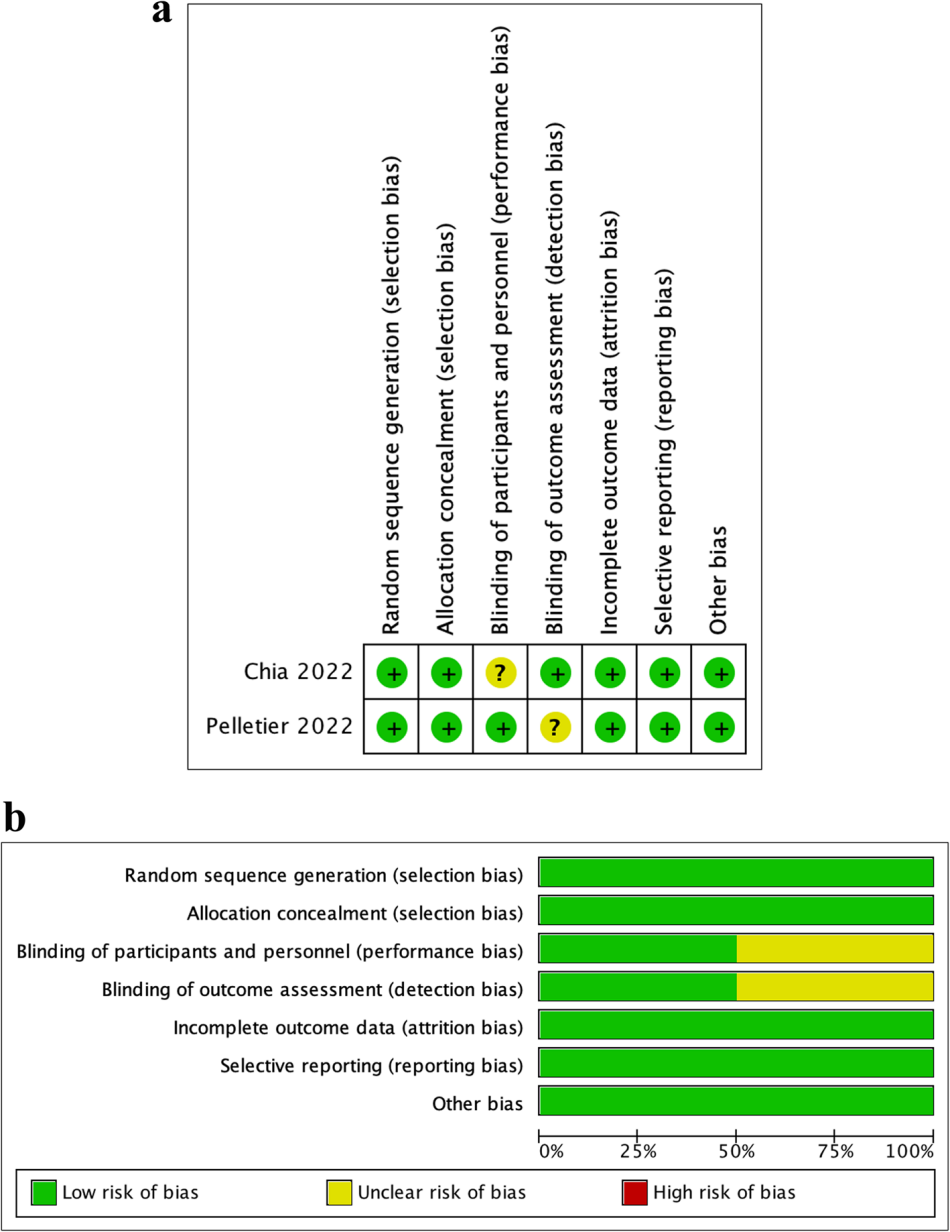
**a** Risk of bias summary of the included RCTs. **b** Risk of bias item presented as percentage across two included RCTs

As was shown in Fig. 2a, both RCTs [17, 23] detailed the generation method of the random sequence and carried out allocation and concealment. The outcome indicators were also reported, and appropriate models were used to process the missing data. The reason for the uncertainty of two bias risks was that Chia et al. did not explain whether to implement the blinding method for the test personnel, while Pelletier et al. did not provide sufficient information to explain whether to implement the blinding method for the outcome evaluators. Figure 2b details the analysis of risk of bias results.

**Fig. 3:**
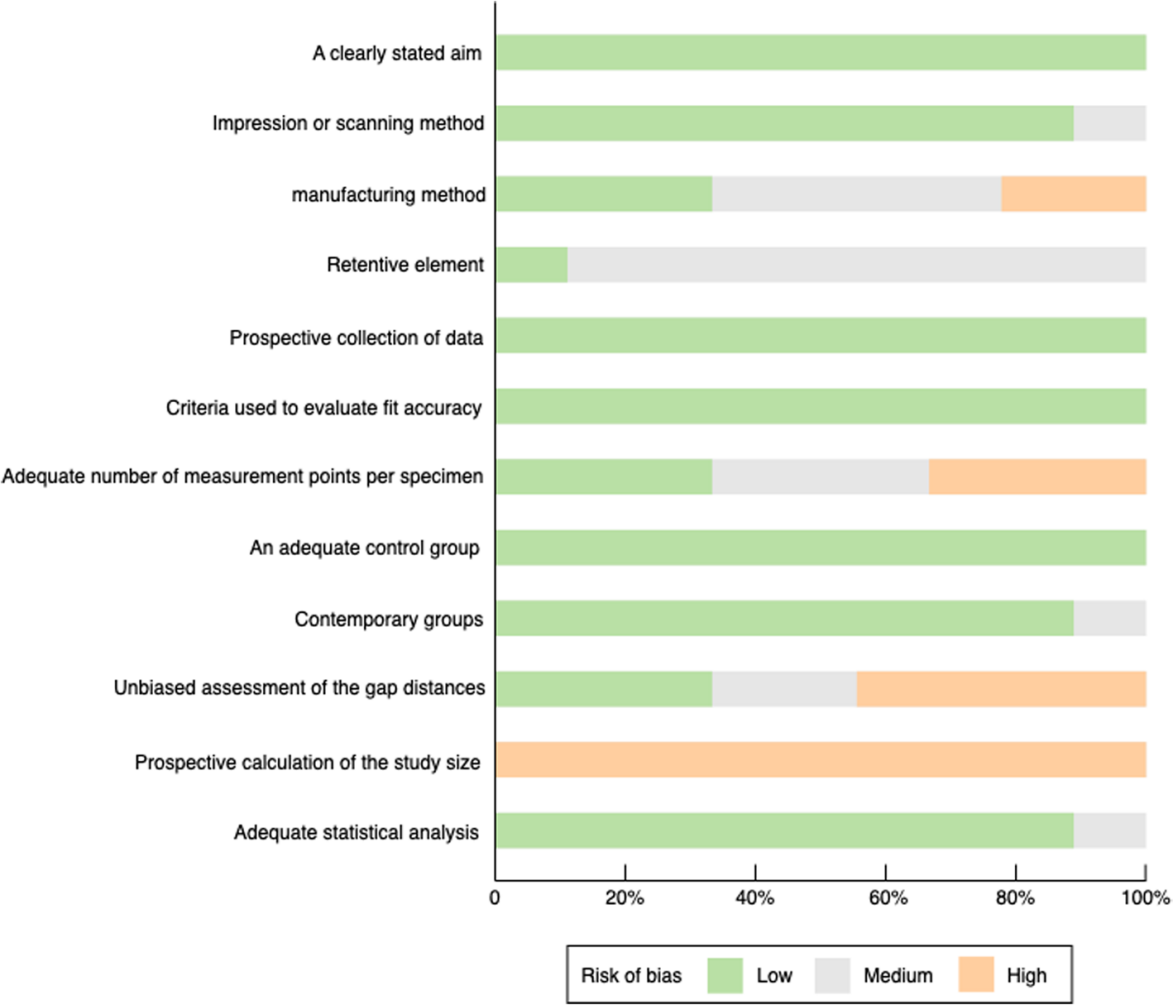
Risk of bias by modified MINORS scale. Author’s judgments about each item presented as percentage across all in vitro studies and a non-randomized clinical study

As shown in Table 7, among the 9 non-randomized studies accessed by modified MINORS scale, 5 demonstrated low risk of bias, and 3 were classified as medium risk of bias, with only 1 presented high risk of bias [12]. This was mainly caused by a lack of information about the number of measurement sites for each sample, and the outcome data of control group was retrieved from their previous study. All the studies clearly stated their aims and made quantitative evaluation of fit accuracy. An adequate control group was set in both clinical and in vitro studies. One item, viz. prospective calculation of the study size, was of high risk of bias, with none of the included studies reporting on this item (Fig. 3).

**Table 7:** Evaluation of risk of bias by modified MINORS scale

Methodological index for included studies	In Vitro Study									Clinical Study
	Arnold et al., 2018 [13]	Bajunaid et al., 2019 [20]	Nakata et al., 2017 [11]	Ye et al., 2018 [19]	Soltanzadeh et al., 2019 [24]	Torii et al., 2018 [12]	Oh et al., 2022 [22]		Muehlemann et al., 2022 [21]	Ye et al., 2017 [18]
A clearly stated aim	2	2	2	2	2	2	2		2	2
Impression or scanning method	2	2	2	2	2	2	2		1	2
Manufacturing method	1	1	2	0	0	1	2		2	1
Abutment	1	1	1	1	1	1	1		1	2
Prospective collection of data	2	2	2	2	2	2	2		2	2
Criteria used to evaluate fit accuracy	2	2	2	2	2	2	2		2	2
Adequate number of measurement points per specimen	2	2	0	0	2	0	1		1	1
An adequate control group	2	2	2	2	2	2	2		2	2
Contemporary groups	2	2	2	2	2	1	2		2	2
Unbiased assessment of the gap distances	1	1	0	2	2	0	2		0	0
Prospective calculation of the study size	0	0	0	0	0	0	0		0	0
Adequate statistical analysis	2	2	2	1	2	2	2		2	2
Additional criteria for included clinical studies										
Baseline equivalence of groups	/	/	/	/	/	/		/	/	2
Total Score	19	19	17	16	19	15		20	17	20
Risk of bias	Low	Low	Medium	Medium	Low	High		Low	Medium	Low

0: not reported, 1: reported but inadequate, 2: reported and adequate; The global ideal score being 24 for in vitro studies and 26 for clinical studies. Risk of bias of individual study judged by total score: < 16 high, 16–18 medium, > 18 low

### Results of syntheses

**Fig. 4:**
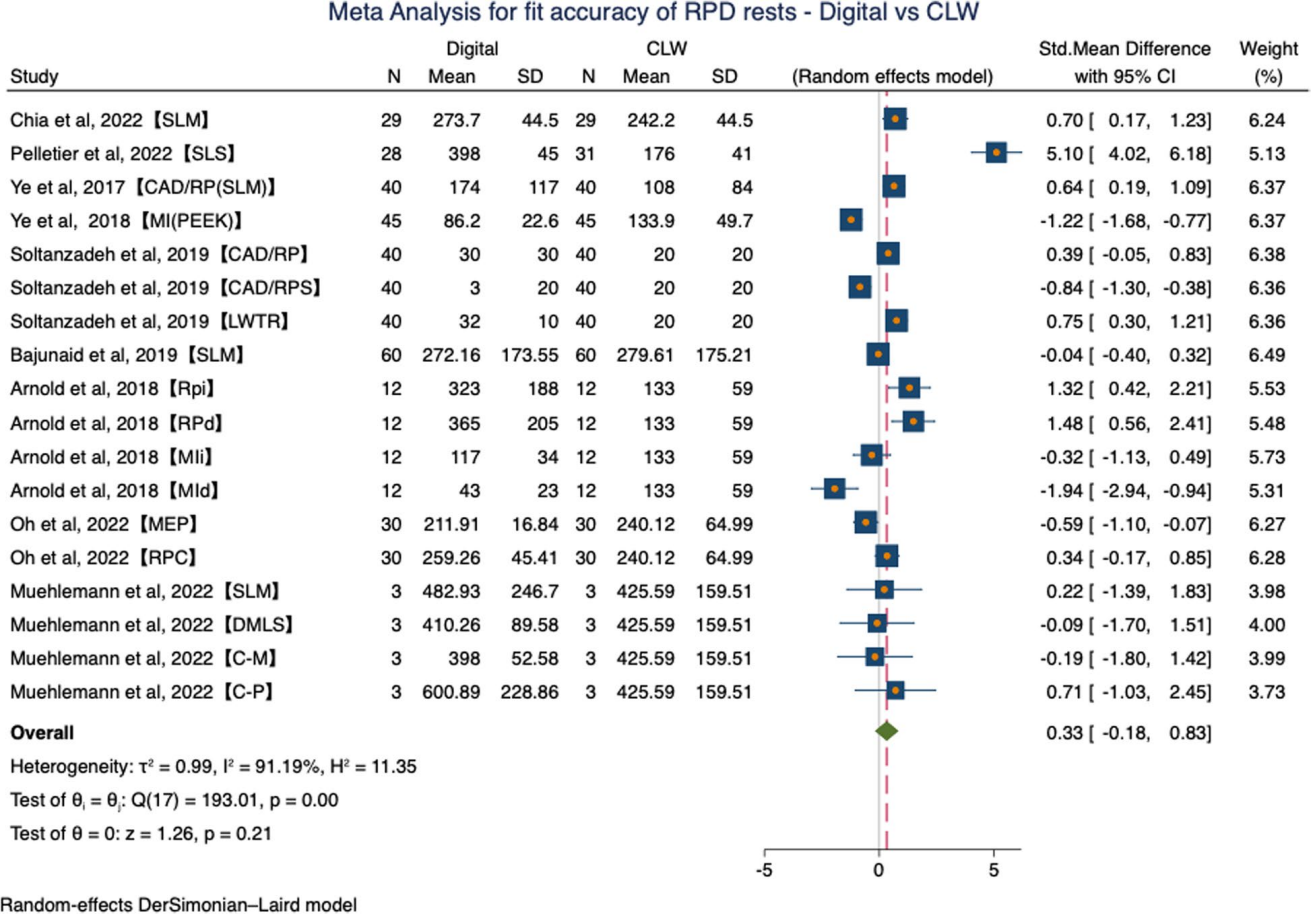
Forest plot of meta-analysis for fit accuracy of RPD rests – Digital vs CLW

There are three studies with missing outcome data. We contacted the authors, but only one responded [21], leaving the two remaining studies to be excluded from the meta-analysis [11, 12]. In the global meta-analysis performed, the SMD was 0.33(95%CI: -0.18, 0.83, *P* = 0.21) in favor of CLW. But this difference did not show statistical significance (*P* > 0.05) and high statistical heterogeneity was found (τ^2^ = 0.99, I^2^ = 91.19%, H^2^ = 11.35, Random effects model) (Fig. 4).

### Sensitivity analysis

**Fig. 5:**
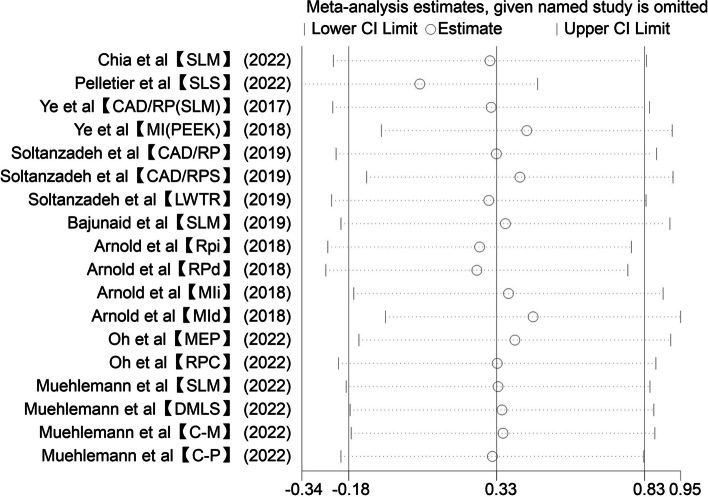
Sensitivity analysis – Digital vs CLW

**Fig. 6:**
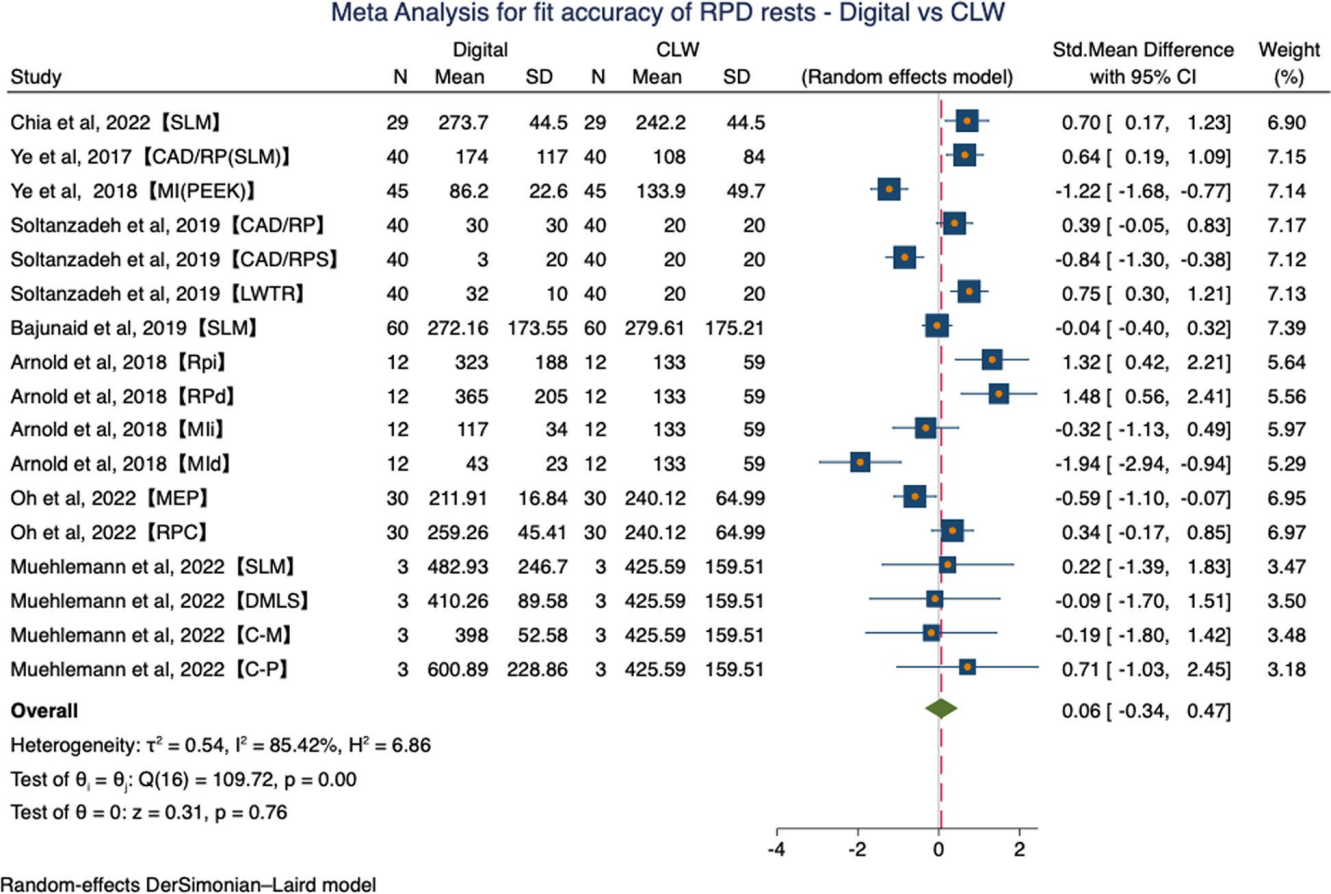
The result of sensitivity analysis (after removing the study of Pelletier)

The result of the sensitivity analysis is shown in Fig. 5, indicating that research done by Pelletier had the most effect on heterogeneity, and heterogeneity decreased after removing this study [I^2^ = 85.42%, SMD = 0.06, 95%CI (-0.34, 0.47), *P* = 0.76] (Fig. 6).

### Subgroup analysis

**Fig. 7:**
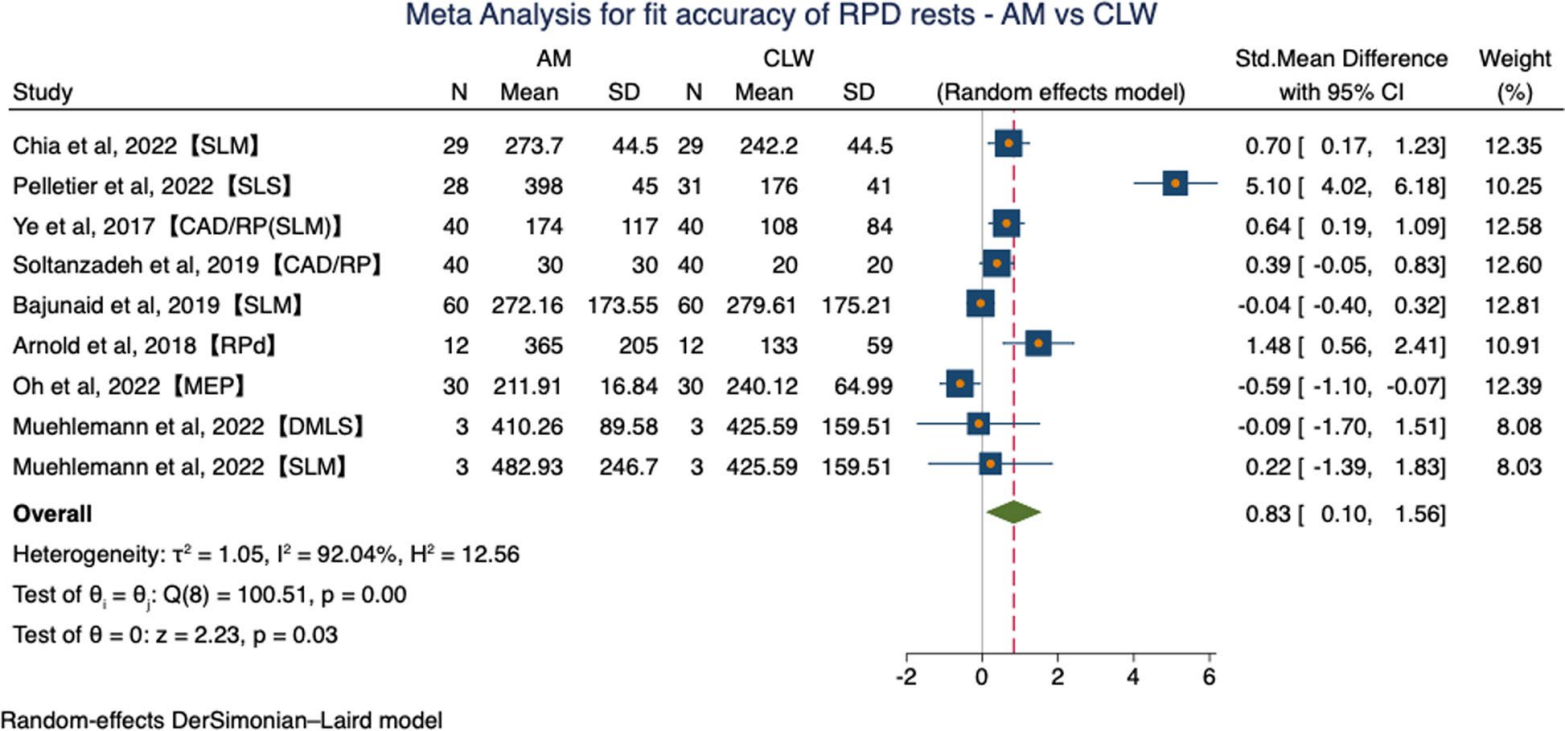
Forest plot of meta-analysis for fit accuracy of RPD rests—AM vs CLW

Subgroup analysis was conducted for different types of digital technologies. All studies were compared according to three groups (Table 8). A comparison of fit accuracy in the rest region between AM RPDs and CLW ones involving 9 groups from 8 studies was performed, which showed significant difference between AM and CLW (SMD = 0.83, 95%CI (0.10, 1.56), *P* = 0.03). Significant heterogeneity between analyses was identified (*P* = 0.00, I^2^ = 92.04%) in a random-effects model (Fig. 7).

**Table 8:** Results of subgroup analysis

Subgroup	Study number	Heterogeneity		Effects model	Meta-analysis		
		I^2^	*P*		SMD	95%CI	*P*
AM VS CLW	9	92.04%	0.00	Random	0.83	(0.10, 1.56)	0.03
MI VS CLW	2	38.73%	0.20	Fixed	-1.35	(-1.76, -0.93)	0.00
Indirect VS CLW	6	47.22%	0.09	Fixed	0.51	(0.23,0.80)	0.00

**Fig. 8:**
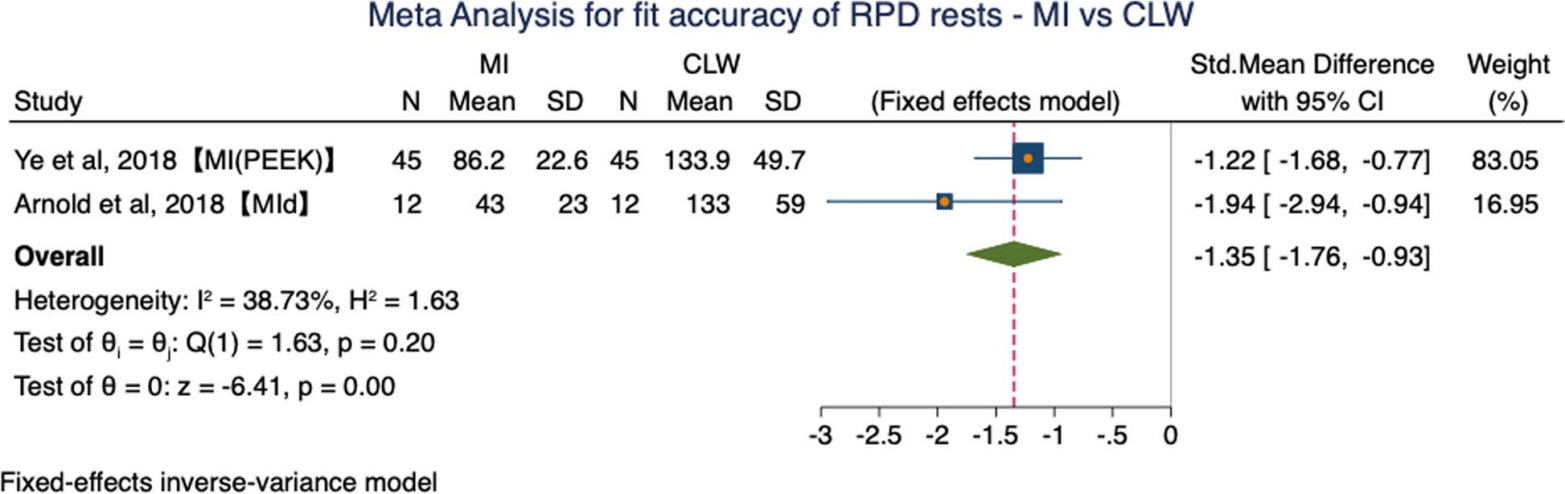
Forest plot of meta-analysis for fit accuracy of RPD rests—MI vs CLW

While in subgroup (MI vs CLW), there was a statistically significant difference with a favorable trend in the MI technique (*P* = 0.00 < 0.05) (Fig. 8). SMD was -1.35 (95% CI: -1.76 to -0.93) and low heterogeneity was identified (*P* = 0.20; I^2^ = 38.73%, fixed effects model) (Fig. 8).

**Fig. 9:**
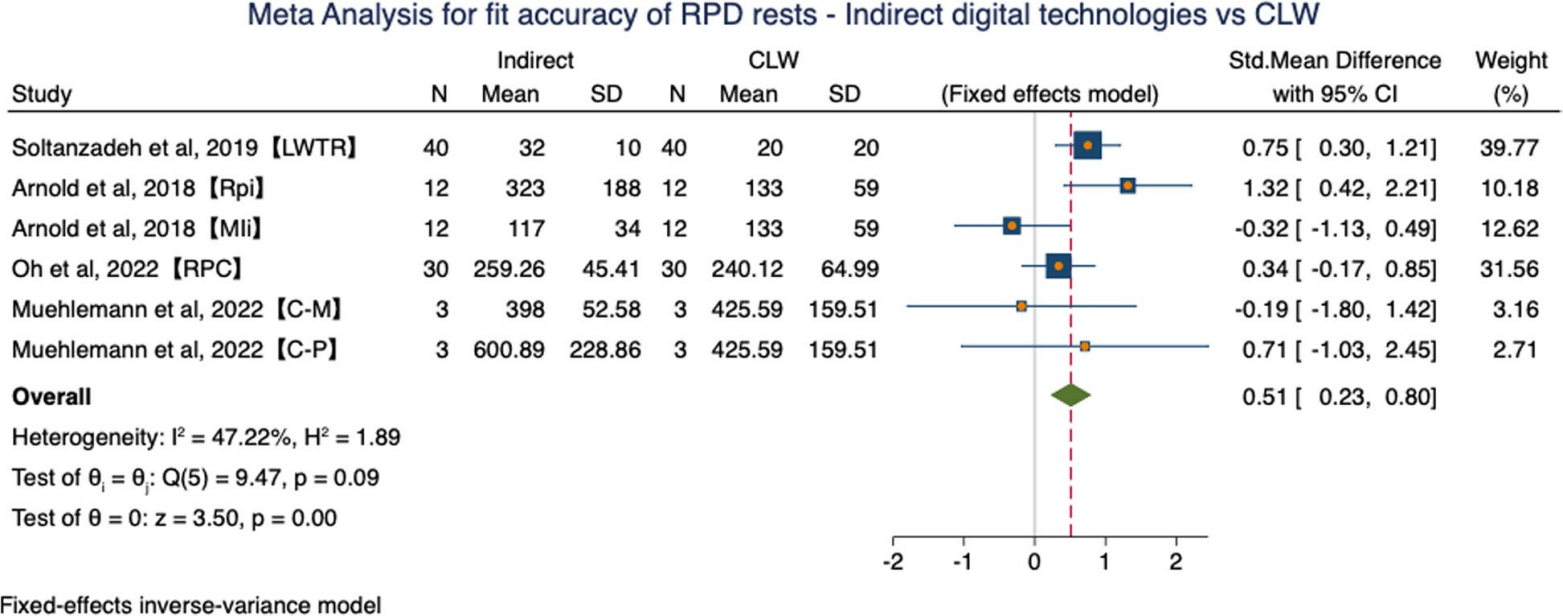
Forest plot of meta-analysis for fit accuracy of RPD rests – Indirect digital technologies vs CLW

The subgroup of Indirect digital technologies vs CLW included 6 groups from 4 studies. Results in a fixed effects model indicated that CLW RPDs obtained a significant better fit accuracy in rest region than RPDs fabricated by indirect digital technologies (SMD = 0.51, 95%CI (0.23, 0.80), *P* = 0.00). Low heterogeneity between these analyses was identified (*P* = 0.09, I^2^ = 47.22%) (Fig. 9).

**Fig. 10:**
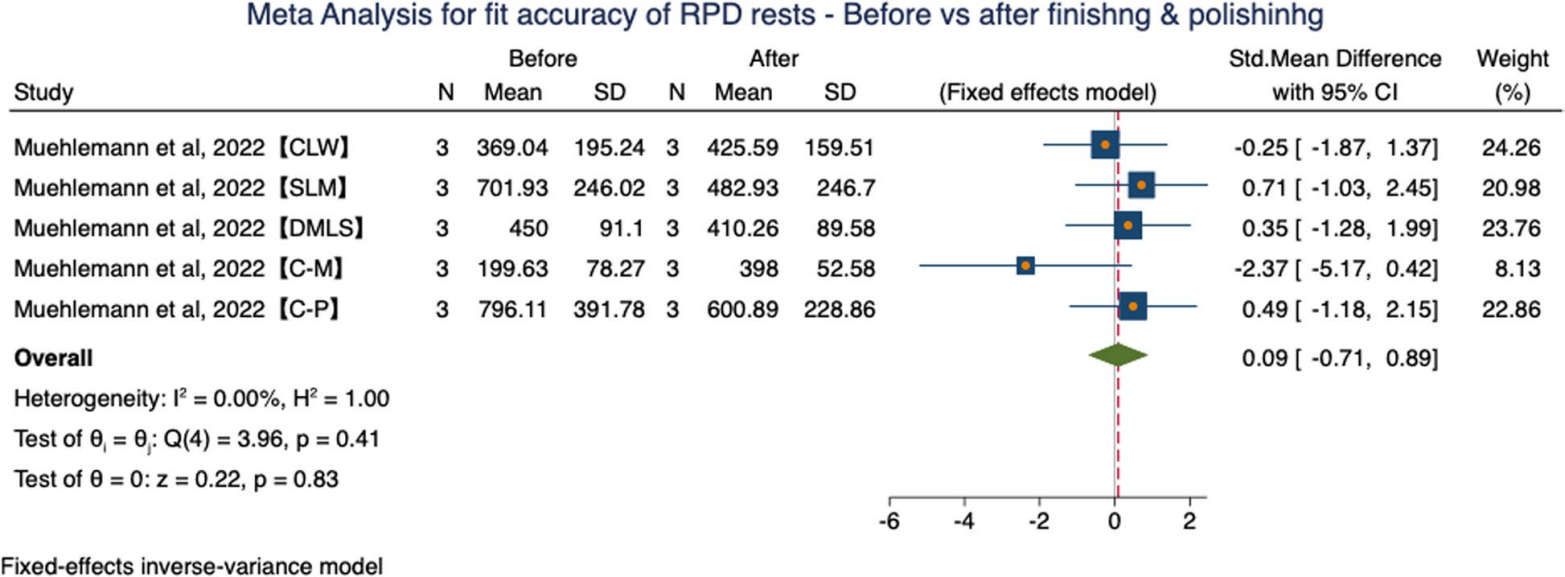
Meta-analysis for fit accuracy of RPD rests—before vs after finishing & polishing

One study evaluated the fit accuracy of RPD rest in all groups before and after finishing and polishing [21]. To evaluate the potential effect of finishing and polishing procedure to the fit accuracy of RPDs, an additional comparison was made and results were presented in Fig. 10. No significant differences between groups were observed (SMD = 0.09, 95%CI (-0.71, 0.89), *P* = 0.83 > 0.05) in a fixed effects model.

Hybrid manufacturing was used in two in vitro studies [11, 12] and compared with CLW. Both studies fabricated Aker’s clasp assemblies by the same one-process molding machine. And silicone film method was used to measure the gap distance between the clasp samples and the stainless-steel model. Since part of the outcome data was not provided in the form of Mean ± Standard Deviation and contact was not responded, they were excluded from meta-analysis. The outcome data were extracted from the histograms and box plots provided in the original articles with the assistance of a software program (GetData Graph Digitizer version 2.26.0.20). The results are presented in Table 9.

**Table 9:** Results of two studies included in qualitative description

Author Year	N	Study group	Control group	*p*
Nakata et al., 2017 [11]	15	167.4 ± 9.47μm	123.8 ± 2.93μm	*P* < 0.05
Torii et al., 2018 [12]	20	73.9 ± 1.6 μm	123.8 ± 2.93μm	*P* < 0.05

Nakata et al. reported that compared to cast clasps, the CAM clasps presented significantly greater gap distances (*P* < 0.05) [7]. With digital relief and heat treatment, Torri et al. fabricated HM clasps with better fit accuracy in the rest region [12].

### Reporting biases

**Fig. 11:**
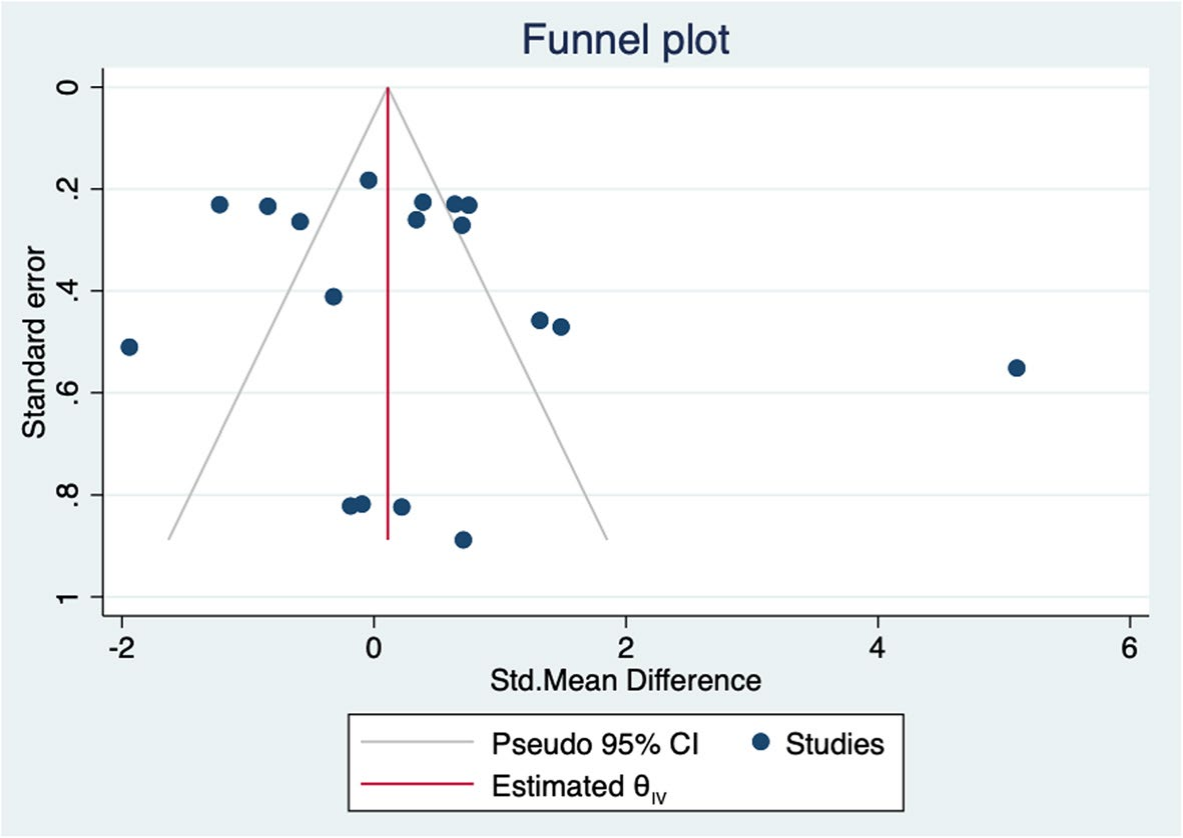
Funnel plot

A funnel plot was constructed to assess publication bias. As shown in Fig. 11, the funnel plot was visually asymmetric, which indicates that potential publication bias may exist.

### Certainty of evidence

**Fig. 12:**
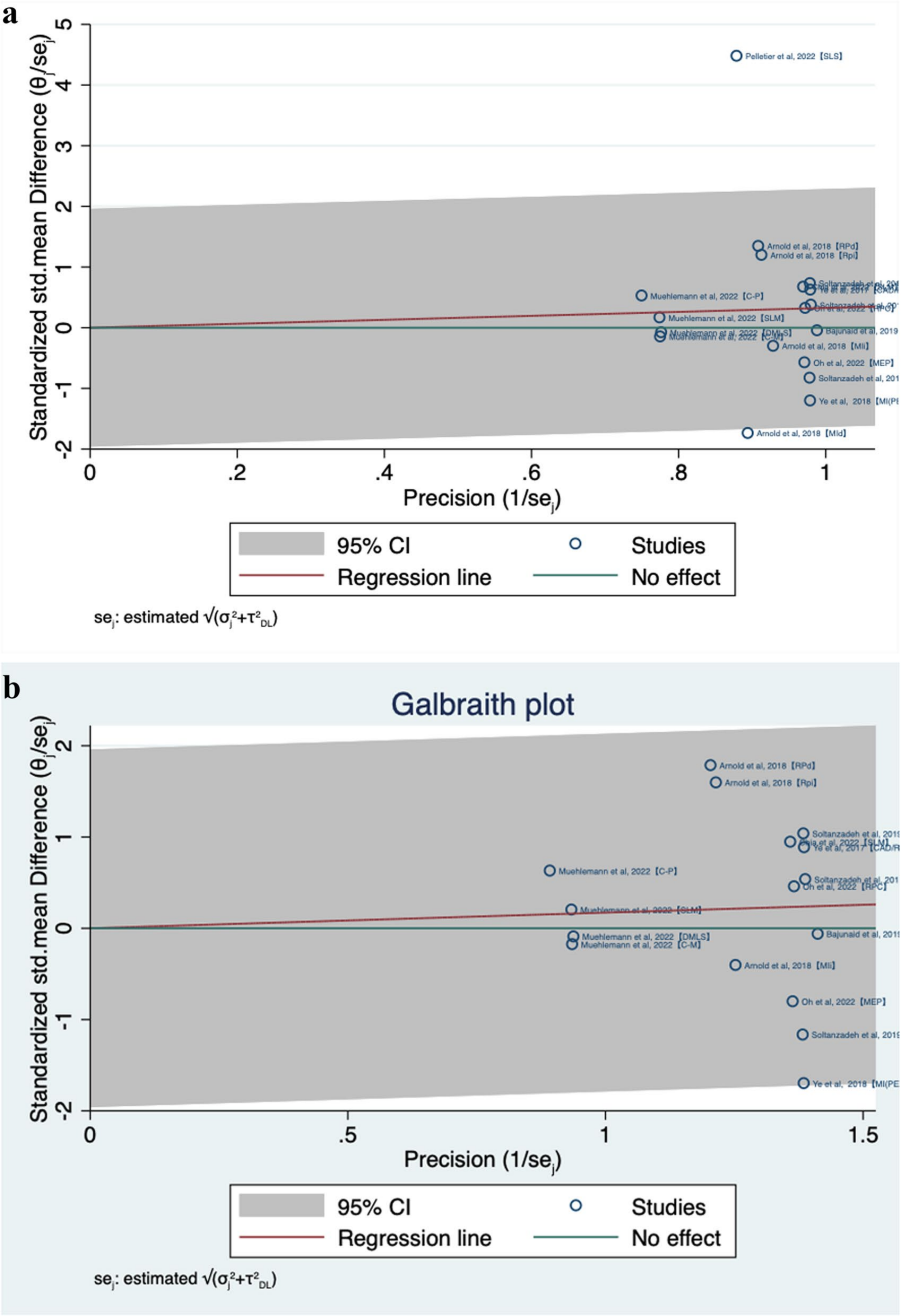
**a** Galbraith plot. **b** Galbraith plot

As the Galbraith plot shows, most of the points which represent individual studies were within the range of the 95% CI regression line (Fig. 12a) except for two studies: Pelletier et al. [23]【SLS】and Arnold et al. [13]【MId】. After excluding these two, all of the remaining studies were within the range of the regression line (Fig. 12b), indicating that these two studies may have some impact on the overall effect.

### Fi-index tool

This manuscript has been checked with the Fi-index tool and obtained a score of 0 for the first author only on the date 28/07/2023 according to SCOPUS® [30, 31]. The fi-index tool aims to ensure the quality of the reference list and limit any auto-citations.

## Discussion

The results observed in this study suggested that there were no significant differences in the fit accuracy of the rests of RDPs fabricated by digital technologies and CLW, which means digital technologies can be a viable alternative for the manufacture of RPD frameworks. Subgroup analysis on different types of digital technologies showed that RPDs fabricated by CLW fit significantly better in rest region than those made by AM technologies. Similar result was also found between indirect digital technologies and CLW. However, MI fabricated RPDs presented a significant better fit accuracy in rest region than CLW RPDs. In regard to the effect of finishing and polishing procedure, RPDs before finishing and polishing presented nominally better but not statistically significant fit accuracy in rest region than those after finishing and polishing. With digital relief and heat treatment, HM clasps also presented significantly better fit accuracy in rest region than cast ones [12]. However, this evidence remains to be verified since the HM clasp data was compared to the cast Co-Cr clasp data from their previous study [11] rather than including an in-study control group.

Digital technology developed rapidly in dentistry. With the assistance of the computer, previously manual tasks are becoming faster and easier and the processing costs are reduced as well [5]. However, Takaichi et al. reported that the fitness of the SLM frameworks and clasps was no better than that of cast ones [32]. Moreover, Pordeus et al. reported a similar fit between CAD-CAM technology and the conventional technique [29]. The present results in this study are in agreement with these previous findings [29, 32]. Tan et al. reported that MI is an alternative method of CLW for fabricating titanium RPD clasps [33]. Several other studies have also proved that milled RPDs are comparable to or better than CLW RPDs and thus can be recommended for longer-term clinical use [13, 33, 34]. Results of subgroup analysis for MI vs CLW in this study provides further evidence for this. On the contrary, AM group showed significantly worse fit accuracy compared to CLW group in this review. This finding was corroborated by Pelletier et al. [23] who found that SLS frameworks exhibited significantly worse clinical accuracy as well as higher variability at the rest region than CLW frameworks. Arnold et al. also reported distinct fitting irregularities in the fit of RPDs fabricated with RP techniques [13].

In a previous study, Michael Braian found out that among the five AM units namely Arcam®, Concept laser®, EOS®, SLM Solutions® and EOS®(Co-Cr), the highest overall fabrication precision was achieved by EOS (CoCr) which was below 0.050 mm, close to that of SM system (Mikron®) [4]. While the other AM machines presented just acceptable precision (< 0.150 mm) on all axes except for the z-axis, which was even worse (> 0.5 mm) [4]. It can be inferred that different AM machines as well as different parameters can affect the fit accuracy of the end product [4], which may also be a possible explanation for the high heterogeneity in the pooled result and in AM subgroup. These results demonstrate that AM techniques should be further improved and standardized in RPD fabrication to make sure that every framework is produced with consistent accuracy [4]. Nonetheless, with higher fabrication speed and better accuracy, additive manufacturing will seriously compete with traditional manufacturing in creating good end-use products [1, 5].

Except for the 11 included studies, many other studies evaluated the fit accuracy of digitally fabricated RPDs [34–39]. These studies, because they set no control group [35–37] or performed indirect digital technologies as control groups [34, 38, 39] were excluded after screening. As far as indirect digital technologies are concerned, before conventional investment casting, digital model is obtained by scanning and computer-aided design is performed followed by printing or milling of wax or resin pattern [34, 38, 40]. Therefore, in this study, these indirect technologies were not included within the scope of conventional method and were taken as digital technology for RPD fabrication. A comparison between indirect digital technologies and CLW reflects the difference between digital scanning and conventional method of impression-taking and working cast fabrication, while a comparison between indirect digital technologies and fully digital workflow represents the processing tolerance produced from investment to finishing. However, what is really significant in clinical practice is a summation of the errors involved in all the steps from the scanning to the post-treatment process and also in all the stages of CLW from impression taking to finishing and polishing. For these reasons, a universal classification of RPD fabrication technologies is suggested, especially for indirect methods.

Sensitivity analyses showed that another possible cause of heterogeneity was the measurement method. Similar result was reported by Alabdullah et al., who compared the different approaches to evaluating the fit of RPD frameworks and concluded that the discrepancies in the gap distance values are likely to be caused by different registration methods [41]. There is no gold standard for assessing the fit accuracy of RPD. Quantitative methods include silicon film method and surface-matching, and the latter can be carried out whether by matching the surface from the master model and the master model with the silicone registration attached [22, 42] or superimposing the intaglio surfaces of RPD frameworks onto the STL file of the master model [24]. Besides, the direct optical observation was also used to analyze the fit accuracy by light microscopy [13, 43, 44]. Silicone film method is commonly performed by inserting silicone impression material between the RPD and intraoral dentition or master model under a retentive force that is maintained through the setting time. The the silicone replica of the gap may be sectioned afterwards and its thickness directly revealing the gap was measured with stereomicroscope, digital microscope, electronic calipers or profile projector [18, 35, 45, 46]. However, Yoon et al. reported that the number of measuring points have effect on the average thickness of the silicon replicas, and that the accuracy of silicone film method was not sufficiently reliable [47]. In contrast, three-dimensional surface-matching can be used to assess the fit accuracy of RPDs more comprehensively and effectively than silicone film method [47]. Consequently, for silicone film method, the adequate force applied during the setting time, the type of silicone replica, as well as the number and site of measuring points need to be clarified, which is necessary to insure the reliability and reproducibility of the outcomes of individual studies in the future.

In addition, up to now there is no consensus about the clinical acceptable gap distance of RPDs. Stern et al. reported that a gap of 0 to 50 μm was deemed to be close contact [16]. In a clinical study conducted by Dunham et al., the average gap distance between the rests and the rest seats was 193 ± 203 μm, ranging from 0 to 828 μm [48]. Li et al. fabricated 13 one-piece PEEK RPDs and the gap distance was 84.3 ± 23.6 µm in rest region [37]. Among the present 11 included studies, the mean average gap distances in rest region ranged from 30 μm to 365 μm in digitally fabricated RPDs, and 20 μm to 279.61 μm in CLW groups. Several studies compared the overall fit accuracy of RPD frameworks fabricated by digital and conventional technologies [19, 24, 35, 42], and some only evaluated the fit accuracy of clasps [11, 12, 34, 40, 43]. However, the low overall internal discrepancy value is not equivalent to better fit, as it is the compounded result of individual components. The RPD rests could be the ideal reference for the fit evaluation, which is easy for measurement and important for functional loading of the overall framework [45]. The fit accuracy of other RPD components, namely the connectors, the clasp arms as well as denture base should be further investigated for both digital and conventional fabrication technologies.

One of the factors that is most influential for fit accuracy is the finishing and polishing procedures on the tissue surface of the frameworks, especially for the rests [20]. In other words, to improve fit accuracy, finishing procedures in the laboratory should be well-controlled and excessive removing of metal from the intaglio surface should be avoided [16]. Most of the RPD samples included in this review were polished [13, 17, 18, 20–23], except in 3 studies [11, 12, 24]. Different methods and extent of manual polishing are likely to affect the interpretation of the final results, while meta-analysis in this review showed no significant influence of finishing and polishing on the fit accuracy in rest region. Hence, further studies considering finishing and polishing procedure with a larger sample size are needed to validate this conclusion.

### Limitation

Only 11 studies were included in this meta-analysis after a comprehensive search in the main databases, indicating that the number of original research in relevant fields is still limited. No consensus has been reached for quality assessment of in vitro studies. The modified version of MINORS scale has not been validated in terms of index content as well as scoring, and the results of assessment can only be referenced conservatively. Moreover, since the findings of this review are mainly based on in vitro studies, caution must be exercised when applying the results into clinical practice.

## Conclusions

RPDs fabricated by digital technologies exhibit comparable fit accuracy in rest region with those made by CLW.
A universal classification of RPD fabrication workflow is suggested especially for indirect digital methods.
3.Standardizing the measurement method and setting specific values of fit evaluation of RPDs are two important tasks at current research as well as clinical practice.

### Supplementary Information

**Additional file 1.**

## Data Availability

All data generated and analysed during this study are included in this published article [and its supplementary information files].

## Abbreviations

RPDs: Removable partial dentures
CLW: Conventional lost-wax technique
MINORS: Modified methodological index for non-randomized studies
RCTs: Randomized controlled trials
SMD: Standardized mean difference
CI: Confidence intervals
AM: Additive manufacturing
HM: Hybrid manufacturing
CAD/CAM: Computer-aided design/computer-aided manufacturing
SM: Subtractive manufacturing
CNC: Computer Numerical Controlled
RP: Rapid prototyping
SLA: Stereolithography
SLM: Selective laser melting
SLS: Selective laser sintering
DMLS: Direct metal laser-sintering
FDM: Fused deposition modeling
SEBM: Selective electron beam melting
MI: Milling
PRISMA: Preferred Reporting Items for Systematic Review and Meta-Analyses
PICOS: Population, Intervention, Comparison, Outcome and Study
MeSH: Medical Subject Headings
MINORS: Methodological Index for Non-Randomized Studies
Co-Cr: Cobalt-chromium
PEEK: Polyetheretherketone
RLS: Repeated laser sintering
CAD/RPS: Selective laser melting from stone model
LWTR: Lost-wax technique from resin model
Rpi: Indirect rapid prototyping
Rpd: Direct rapid prototyping
MIi: Indirect milling
MId: Direct milling
MEP: Metal 3D printing
RPC: Resin printing and subsequent casting
C-M: Conventional casting of milled sacrificial patterns
C-P: Conventional casting of printed sacrificial patterns
CON: Conventional lost-wax technique
NR: Not reported
