# Supplementary material for: Fit accuracy in the rest region of RPDs fabricated by digital technologies and conventional lost-wax casting: a systematic review and meta-analysis
Source: BMC Oral Health. 2023 Sep 15;23:667. doi: 10.1186/s12903-023-03348-6 (PMC10504730; doi:10.1186/s12903-023-03348-6)
Supplement: Supplementary file 1 — Additional file 1. [file 12903_2023_3348_MOESM1_ESM.pdf]

NHS
National Institute for Health Research

UNIVERSITY of York
Centre for Reviews and Dissemination

Systematic review

A list of fields that can be edited in an update can be found here

1. * Review title. [1 change]

Give the title of the review in English

Evaluation of fit accuracy in the rest region of removable partial dentures (RPDs) fabricated by digital technologies: A systematic review and meta-analysis

2. Original language title.

For reviews in languages other than English, give the title in the original language. This will be displayed with the English language title.

3. * Anticipated or actual start date.

Give the date the systematic review started or is expected to start.

28/07/2020

4. * Anticipated completion date.

Give the date by which the review is expected to be completed.

28/07/2021

5. * Stage of review at time of this submission. [1 change]

This field uses answers to initial screening questions. It cannot be edited until after registration.

Tick the boxes to show which review tasks have been started and which have been completed.

Update this field each time any amendments are made to a published record.

The review has not yet started: No

Review stage	Started	Completed
Preliminary searches	Yes	No
Piloting of the study selection process	Yes	No
Formal screening of search results against eligibility criteria	Yes	No
Data extraction	No	No
Risk of bias (quality) assessment	No	No
Data analysis	No	No

Provide any other relevant information about the stage of the review here.

6. * Named contact.

The named contact is the guarantor for the accuracy of the information in the register record. This may be any member of the review team.

Jiachao Qiu

Email salutation (e.g. "Dr Smith" or "Joanne") for correspondence:

Miss Qiu

7. * Named contact email.

Give the electronic email address of the named contact.

gladys_tmu66@163.com

8. Named contact address

Give the full institutional/organisational postal address for the named contact.

No.22 Qixiangtai Road, Heping District, Tianjin 300070, China

9. Named contact phone number.

Give the telephone number for the named contact, including international dialling code.

86-22-23332080

10. * Organisational affiliation of the review. [1 change]

Full title of the organisational affiliations for this review and website address if available. This field may be completed as 'None' if the review is not affiliated to any organisation.

Department of Prosthodontics, School & Hospital of Stomatology, Tianjin Medical University, Tianjin 30070, China

Organisation web address:

11. * Review team members and their organisational affiliations. [1 change]

Give the personal details and the organisational affiliations of each member of the review team. Affiliation refers to groups or organisations to which review team members belong. **NOTE: email and country now MUST be entered for each person, unless you are amending a published record.**

Miss Jiachao Qiu. Department of Prosthodontics, School & Hospital of Stomatology, Tianjin Medical University, Tianjin 30070, China
Miss Danfeng Wu. Department of Prosthodontics, School & Hospital of Stomatology, Tianjin Medical University, Tianjin 30070, China
Dr Wei Liu. Department of Prosthodontics, School & Hospital of Stomatology, Tianjin Medical University, Tianjin 30070, China
Dr Lei Sui. Department of Prosthodontics, School & Hospital of Stomatology, Tianjin Medical University, Tianjin 30070, China

12. * Funding sources/sponsors.

Details of the individuals, organizations, groups, companies or other legal entities who have funded or sponsored the review.

This study was supported by the National Natural Science Foundation of China (NSFC)

Grant number(s)

State the funder, grant or award number and the date of award

81970958

13. * Conflicts of interest.

List actual or perceived conflicts of interest (financial or academic).

None

14. Collaborators.

Give the name and affiliation of any individuals or organisations who are working on the review but who are not listed as review team members. **NOTE: email and country must be completed for each person, unless you are amending a published record.**

15. * Review question. [1 change]

State the review question(s) clearly and precisely. It may be appropriate to break very broad questions down into a series of related more specific questions. Questions may be framed or refined using PI(E)COS or similar where relevant.

This study aims to analyze the fit accuracy in the rest region of removable partial dentures (RPDs). For RPDs, are digital technologies more effective compared to conventional lost-wax (CLW) technique in terms of fit accuracy in the rest region?

16. * Searches. [1 change]

State the sources that will be searched (e.g. Medline). Give the search dates, and any restrictions (e.g. language or publication date). Do NOT enter the full search strategy (it may be provided as a link or attachment below.)

The following electronic sources will be searched: Web of Science, PubMed, EMbase and Cochrane Library. No date or publication language limits are taken into account. Studies conducted between January 1950 until July 2020 will be included. Other popular online internet search engines e.g. Google, Yahoo, Wiley Online Library and some regional electronic bibliographic databases including WANFANG DATA (http://www.wanfangdata.com.cn/index.html) and CNKI(https://www.cnki.net) will also be searched. A supplementary manual search will be carried out on eligible articles. The searches will be re-run just before the final analyses and further studies retrieved for inclusion in this review.

17. URL to search strategy. [1 change]

Upload a file with your search strategy, or an example of a search strategy for a specific database, (including the keywords) in pdf or word format. In doing so you are consenting to the file being made publicly accessible. Or provide a URL or link to the strategy. Do NOT provide links to your search **results**.

Alternatively, upload your search strategy to CRD in pdf format. Please note that by doing so you are consenting to the file being made publicly accessible.

Do not make this file publicly available until the review is complete

18. * Condition or domain being studied. [1 change]

Give a short description of the disease, condition or healthcare domain being studied in your systematic review.

Removable partial dentures (RPDs) are traditionally fabricated by lost-wax casting, which is a time-consuming and error-prone process. Recently, Digital technologies including additive manufacturing (3D printing etc.), subtractive manufacturing (computerized numerical control milling technology etc.) and hybrid manufacturing have been increasingly gaining their popularities. The first quality of digital technologies is to produce prosthetic components with improved fit accuracy compared with traditional lost-wax technique.

However, there is still a lack of evidence with high quality to prove their efficiency. This review focuses on studies evaluating the fit accuracy in the rest region of RPDs fabricated by digital technologies.

19. * Participants/population.

Specify the participants or populations being studied in the review. The preferred format includes details of both inclusion and exclusion criteria.

Removable partial dentures

20. * Intervention(s), exposure(s). [1 change]

Give full and clear descriptions or definitions of the interventions or the exposures to be reviewed. The preferred format includes details of both inclusion and exclusion criteria.

Digital technologies including additive manufacturing (3D printing etc.), subtractive manufacturing (computerized numerical control milling technology etc.) and hybrid manufacturing.

21. * Comparator(s)/control.

Where relevant, give details of the alternatives against which the intervention/exposure will be compared (e.g. another intervention or a non-exposed control group). The preferred format includes details of both inclusion and exclusion criteria.

Conventional lost-wax (CLW) technique

22. * Types of study to be included. [1 change]

Give details of the study designs (e.g. RCT) that are eligible for inclusion in the review. The preferred format includes both inclusion and exclusion criteria. If there are no restrictions on the types of study, this should be stated.

Included:(1) Randomized controlled trials, prospective or retrospective studies, clinical studies, in vitro and in vivo studies (2) Studies that comparing the fit accuracy of RPD rests fabricated by digital technologies and these of conventional lost-wax (CLW) technique. (3) Articles published in the period from 1950 until July 2020. Excluded: (1) Articles that used only qualitative method to evaluate the quality of fit, such as pressing test and clinical check without available data. (2) Articles that studied the RPDs fabricated by printing or milling wax/resin patterns before casting rather than CLW technique as control groups. (3) Articles unavailable in the databases or those that could not be accessed to read in full.

23. Context.

Give summary details of the setting or other relevant characteristics, which help define the inclusion or exclusion criteria.

24. * Main outcome(s). [1 change]

Give the pre-specified main (most important) outcomes of the review, including details of how the outcome is

defined and measured and when these measurement are made, if these are part of the review inclusion criteria.

Fit accuracy is defined as the gap distance in micrometers between the rest and the its corresponding rest seat area. The gap distance is measured by silicone film method, visual inspection using magnification device or scanning the prosthesis and the cast and superimposing the two STL files of each scan using surface matching software.

Measures of effect

Please specify the effect measure(s) for you main outcome(s) e.g. relative risks, odds ratios, risk difference, and/or 'number needed to treat.

The mean difference and standard deviation of gap distances in micrometers will be taken as measures of effect.

25. * Additional outcome(s).

List the pre-specified additional outcomes of the review, with a similar level of detail to that required for main outcomes. Where there are no additional outcomes please state ‘None’ or ‘Not applicable’ as appropriate to the review

Surface roughness on the internal surface of the RPD rest.

Measures of effect

Please specify the effect measure(s) for you additional outcome(s) e.g. relative risks, odds ratios, risk difference, and/or 'number needed to treat.

The surface roughness (Ra) on the internal surface of the onlay rest of each clasp.

[1 change]
26. * Data extraction (selection and coding).

Describe how studies will be selected for inclusion. State what data will be extracted or obtained. State how this will be done and recorded.

Two authors will analyze the studies independently. Firstly, the duplicate records will be excluded. Secondly, titles and abstracts of the various references will be studied separately. Then some articles will be excluded if they do not correspond to the field of investigation or deal with the research question. Finally, some articles will be excluded if they do not meet inclusion criteria. If in doubt, the full text of each article will be reviewed in an independent manner by two authors in order to determine their eligibility. Any disagreement will be resolved by discussion until consensus is reached or by consulting a third author. The extraction and synthesis of data will be done independently by two authors using Microsoft Excel (Version 16.37). The data extracted from each study will be analyzed and the main information obtained in a standardized way and divided into tables. Missing data or additional study information will be requested by contacting corresponding authors of individual studies. If there is no respondence after three contact attempts, the study

will be excluded from meta-analysis and included in the qualitative aspect of the review.

27. * Risk of bias (quality) assessment.

State which characteristics of the studies will be assessed and/or any formal risk of bias/quality assessment tools that will be used.

Risk of bias for RCTs will be assessed by the Cochrane collaboration tool. For prospective study, the Newcastle-Ottawa scale(NOS) will be used. For non-randomized in vitro studies, the risk of bias will be assessed on the modified MINORS scale (Slim et al., 2003). This scale includes ten headings for in vitro studies and two additional headings for in vivo studies. Each heading is rated from 0 to 2 (0 indicates that the content has not been reported; 1, that the content has been reported inadequately; and 2, that the content has been sufficiently reported)

[1 change] 28. * Strategy for data synthesis.

Describe the methods you plan to use to synthesise data. This **must not be generic text** but should be **specific to your review** and describe how the proposed approach will be applied to your data. If meta-analysis is planned, describe the models to be used, methods to explore statistical heterogeneity, and software package to be used.

We envisage a qualitative and quantitative analysis of the data extracted from selected studies in a standardized manner. Dichotomous data will be analyzed using relative risk (RR) and confidence interval (CI) of 95%. Continuous data will be analyzed using the standardized mean difference (SMD) and confidence interval (CI) of 95%. The contribution weight of each study will be conducted to meta-analysis calculations. For all analyzes significant values will be considered as the value of p 0.05. The software Review Manager 5 (Cochrane Group) will be used for the meta-analysis.

[1 change] 29. * Analysis of subgroups or subsets.

State any planned investigation of ‘subgroups’. Be clear and specific about which type of study or participant will be included in each group or covariate investigated. State the planned analytic approach.

We plan to do Sensitivity tests for subgroup analysis in order to avoid potential heterogeneity. Subgroups will be constituted according to the type of the rest (occlusal rest and incisal rest etc.) and material (Cobalt-chromium, titanium and PEEK) used to fabricate RPDs.

30. * Type and method of review.

Select the type of review, review method and health area from the lists below.

Type of review

Cost effectiveness

No

Diagnostic

No

Epidemiologic

No

Individual patient data (IPD) meta-analysis

No

Intervention

No

Living systematic review

No

Meta-analysis

Yes

Methodology

No

Narrative synthesis

No

Network meta-analysis

No

Pre-clinical

No

Prevention

No

Prognostic

No

Prospective meta-analysis (PMA)

No

Review of reviews

No

Service delivery

No

Synthesis of qualitative studies

No

Systematic review

Yes

Other

No

Health area of the review

Alcohol/substance misuse/abuse

No

Blood and immune system

No

Cancer

No

Cardiovascular

No

Care of the elderly

No

Child health

No

Complementary therapies

No

COVID-19

No

Crime and justice

No

Dental

Yes

Digestive system

No

Ear, nose and throat

No

Education

No

Endocrine and metabolic disorders

No

Eye disorders

No

General interest

No

Genetics

No

Health inequalities/health equity

No

Infections and infestations

No

International development

No

Mental health and behavioural conditions

No

Musculoskeletal

No

Neurological

No

Nursing

No

Obstetrics and gynaecology

No

Oral health

No

Palliative care

No

Perioperative care

No

Physiotherapy

No

Pregnancy and childbirth

No

Public health (including social determinants of health)

No

Rehabilitation

No

Respiratory disorders

No

Service delivery

No

Skin disorders

No

Social care

No

Surgery

No

Tropical Medicine

No

Urological

No

Wounds, injuries and accidents

No

Violence and abuse

No

31. Language.

Select each language individually to add it to the list below, use the bin icon to remove any added in error.

English

There is not an English language summary

32. * Country.

Select the country in which the review is being carried out. For multi-national collaborations select all the countries involved.

China

33. Other registration details.

Name any other organisation where the systematic review title or protocol is registered (e.g. Campbell, or The Joanna Briggs Institute) together with any unique identification number assigned by them. If extracted data will be stored and made available through a repository such as the Systematic Review Data Repository (SRDR), details and a link should be included here. If none, leave blank.

34. Reference and/or URL for published protocol.

If the protocol for this review is published provide details (authors, title and journal details, preferably in Vancouver format)

Add web link to the published protocol.

Or, upload your published protocol here in pdf format. Note that the upload will be publicly accessible.

No I do not make this file publicly available until the review is complete

Please note that the information required in the PROSPERO registration form must be completed in full even if access to a protocol is given.

35. Dissemination plans.

Do you intend to publish the review on completion?

Yes

Give brief details of plans for communicating review findings.?

36. Keywords.

Give words or phrases that best describe the review. Separate keywords with a semicolon or new line. Keywords help PROSPERO users find your review (keywords do not appear in the public record but are included in searches). Be as specific and precise as possible. Avoid acronyms and abbreviations unless these are in wide use.

37. Details of any existing review of the same topic by the same authors.

If you are registering an update of an existing review give details of the earlier versions and include a full bibliographic reference, if available.

38. * Current review status.

Update review status when the review is completed and when it is published.New registrations must be ongoing so this field is not editable for initial submission.

Please provide anticipated publication date

Review_Ongoing

39. Any additional information.

Provide any other information relevant to the registration of this review.

40. Details of final report/publication(s) or preprints if available.

Leave empty until publication details are available OR you have a link to a preprint (NOTE: this field is not editable for initial submission). List authors, title and journal details preferably in Vancouver format.

Give the link to the published review or preprint.
